# Emerging applications at the interface of DNA nanotechnology and cellular membranes: Perspectives from biology, engineering, and physics

**DOI:** 10.1063/5.0027022

**Published:** 2020-12-08

**Authors:** Weitao Wang, D. Sebastian Arias, Markus Deserno, Xi Ren, Rebecca E. Taylor

**Affiliations:** 1 Department of Mechanical Engineering, Carnegie Mellon University, Pittsburgh, Pennsylvania 15213, USA; 2 Department of Physics, Carnegie Mellon University, Pittsburgh, Pennsylvania 15213, USA; 3 Department of Biomedical Engineering, Carnegie Mellon University, Pittsburgh, Pennsylvania 15213, USA; 4 Department of Electrical and Computer Engineering, Carnegie Mellon University, Pittsburgh, Pennsylvania 15213, USA

## Abstract

DNA nanotechnology has proven exceptionally apt at probing and manipulating biological environments as it can create nanostructures of almost arbitrary shape that permit countless types of modifications, all while being inherently biocompatible. Emergent areas of particular interest are applications involving cellular membranes, but to fully explore the range of possibilities requires interdisciplinary knowledge of DNA nanotechnology, cell and membrane biology, and biophysics. In this review, we aim for a concise introduction to the intersection of these three fields. After briefly revisiting DNA nanotechnology, as well as the biological and mechanical properties of lipid bilayers and cellular membranes, we summarize strategies to mediate interactions between membranes and DNA nanostructures, with a focus on programmed delivery onto, into, and through lipid membranes. We also highlight emerging applications, including membrane sculpting, multicell self-assembly, spatial arrangement and organization of ligands and proteins, biomechanical sensing, synthetic DNA nanopores, biological imaging, and biomelecular sensing. Many critical but exciting challenges lie ahead, and we outline what strikes us as promising directions when translating DNA nanostructures for future *in vitro* and *in vivo* membrane applications.

## I. INTRODUCTION

Over the past two decades, DNA nanotechnology has advanced rapidly. Today, the quickly falling cost of artificially synthesized DNA, in conjunction with newer approaches for architecting nanostructures, including scaffolded DNA origami and tile-based methods, have paved the way for a variety of static and dynamic 2D and 3D nanomachines as well as tools for measuring the nanoscale environment with unrivaled precision and specificity. Cell biology and engineering have similarly advanced at an increasingly rapid pace, and the biological significance of the cell membrane has never been more apparent. Acting as a semipermeable barrier between the cell and its external environment, as well as enabling the compartmentalization of cytoplasm into organelles, it is vital for cellular uptake, communication, motility, and a myriad of other vital processes. DNA nanotechnology, with its aforementioned capabilities, therefore, allows both qualitative and quantitative studies with applications impossible to achieve through other means.

DNA nanostructures can be designed to bind and interact with lipid membranes through numerous methods. With the capability to target DNA nanostructures onto, into, and through lipid membranes, DNA nanotechnology has spurred an extensive number of novel applications in this interdisciplinary endeavor to investigate and manipulate the cellular membrane and its components with previously unseen specificity and resolution. However, given the vast scope of each individual field (DNA nanotechnology and lipid membrane biology and mechanics), it can be a difficult subject to approach. Furthermore, it is imperative that the literature of previously conducted research is synthesized and the current capabilities of DNA-based systems framed to fully grasp the future applications that could emerge. Our review aims to address both these tasks and has thus been separated into two halves: an introduction to the principles of DNA nanotechnology and lipid/cell membranes that are prerequisite to entering the field, followed by a review of previous research. In the first half, we briefly survey the most common DNA nanotechnology methods currently in use and explain their underlying principles. Next, we review biophysical and mechanical properties of ideal lipid bilayers that play key roles in numerous applications and discuss aspects in which biological membranes go beyond such idealized systems, including potential challenges in moving to biological systems due to the complexity of the cellular environment. Finally, we review the methods by which DNA nanostructures can be delivered and conjugated to lipid membranes. In the second half of our review, we survey promising research applications in the field, such as membrane sculpting, multicell self-assembly, biomolecular and biomechanical sensing, membrane-bound protein organization, synthetic DNA nanopores, and opportunities enabled by bypassing the cellular membrane.

## II. DNA NANOTECHNOLOGY

### A. Scaffolded assemblies

**FIG. 1.**
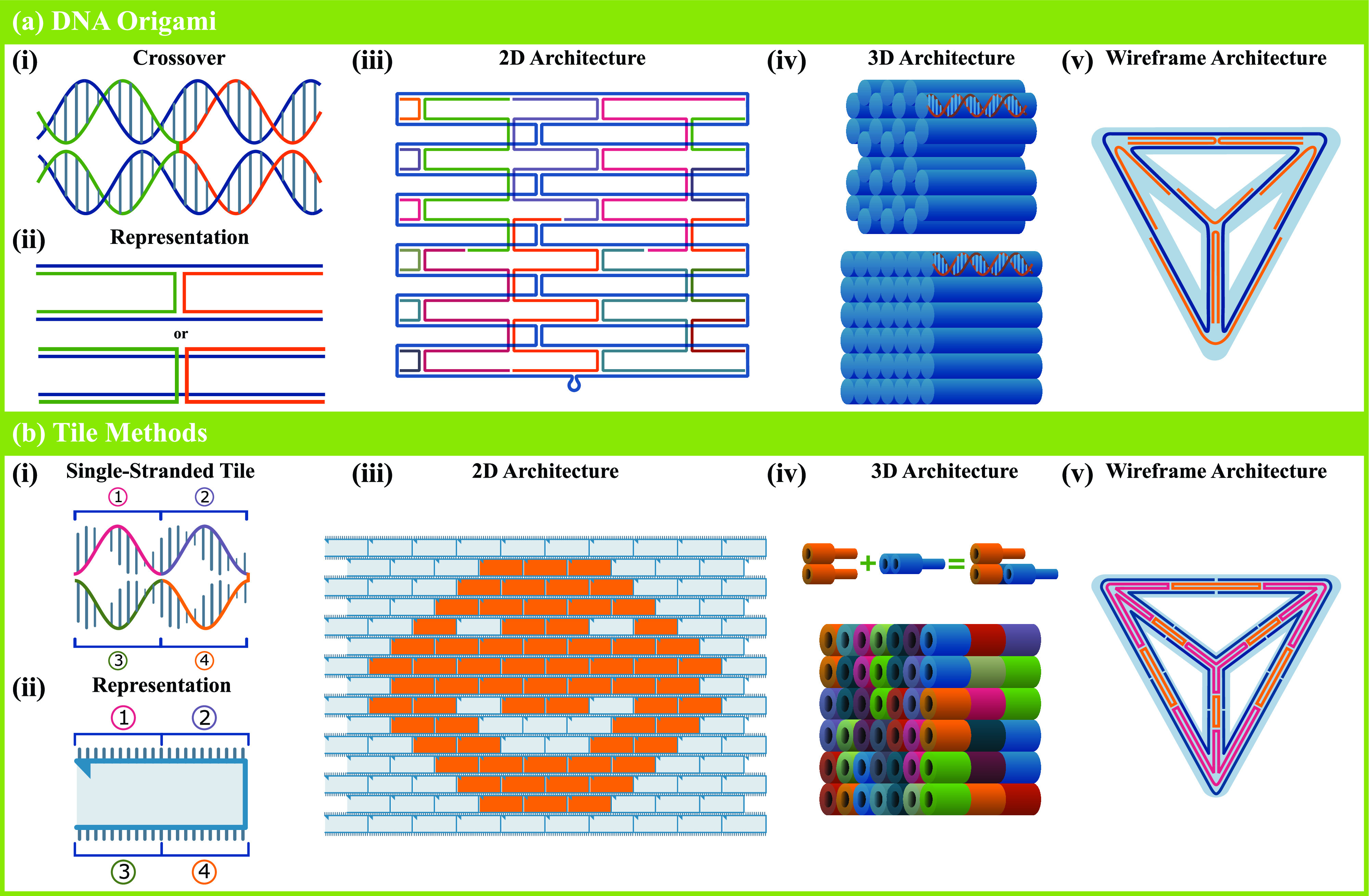
DNA nanotechnology methods. (a) DNA origami. (i) In crossovers, strands running along parallel helices switch to adjacent helices, thereby holding the helices together. Crossovers can only occur when the backbone positions of the helices coincide. (ii) In an abstracted view, DNA strands are represented by lines, where a double-stranded DNA (dsDNA) helix is represented by two adjacent lines. Crossovers are represented by the switching of a strand (line) from one helix to an adjacent one. (iii) In DNA origami constructs, the scaffold strand (blue) is routed in the shape of the desired structure, a rectangle in this case. Staple strands (various colors) are then added to staple the scaffold into the desired shape. The small loop at the bottom represents the unused portion of the scaffold. (iv) 3D DNA origami constructs are generally designed in a square lattice (bottom) or a “honeycomb” hexagonal lattice (top). In these figures, and some subsequent ones, dsDNA helices are depicted as solid cylinders. (v) Wireframe DNA origami tetrahedron design. The scaffold (blue) is routed along the graph representation of a tetrahedron. Staples (orange) serve the same function as in 2D and 3D constructs. This approach can be generalized for more complex wireframe structures. (b) Tile methods. (i) A single-stranded tile is composed of an ssDNA molecule with four regions. Region 1 of the tile will hybridize with region 4 of another and region 2 with region 3 of yet another and vice versa. (ii) Abstract representation of a single-stranded tile. (iii) Hybridization of tiles forms a 2D canvas. Arbitrary shapes can be designed by selecting a subset of canvas, in this case a happy face. (iv) Using the DNA brick method, tile-based 3D structures can be created. In this approach, bricks (shown with various colors) interlock, through strand hybridization, to create a 3D canvas. (v) Tile-based wireframe tetrahedron.

Ned Seeman is credited as the inventor of DNA nanotechnology. Its foundation was laid in the 1980s with the invention of DNA lattices, artificially engineered DNA assemblies in which small DNA complexes hybridize via sticky ends (single stranded overhangs) to create large crystalline structures^1^ followed by the first DNA nanostructure in 1991.^2^ Today, DNA origami stands as one of the most commonly used structural DNA nanotechnology methods. Described in 2006 by Rothemund,^3^ it sets itself apart from previous methods with its ability to create nonperiodic, arbitrarily shaped 2D planar structures with unprecedented complexity (while still retaining the ability to create periodic structures). Borrowing the term “origami,” which describes the Japanese art of paper folding, the basis of this method involves the “folding” of a large single stranded DNA (ssDNA) molecule into a desired shape. It requires a “scaffold” strand, frequently chosen to be the circular single-stranded viral M13mp18 genome as it is a common and cost-effective option (although the use of custom DNA scaffolds has also been demonstrated^4–6^) and a set of hundreds of “staple” strands. The sequence of each staple strand is designed to be complementary to precise regions along the scaffold, with the scaffold coverage percentage dictated by the size of the desired structure. When hybridization occurs, staple strands crimp different sections of the scaffold together using a double-crossover motif, see Fig. 1(a). Staple-scaffold hybridization, therefore, drives the self-assembly of DNA origami with the “building instructions” for any particular structure contained within the staple strand sequences. For this reason, it is rather straightforward to replicate previously formed structures as all that is needed is a list of the required staples. Beyond the planar designs originally demonstrated by Rothemund, three-dimensional (3D) assemblies have been designed and realized by joining planar faces to form cage-like structures,^7,8^ by packing DNA helices in honeycomb, square, and hybrid lattices to create solid 3D objects,^9–11^ and by creating wireframe structures.^12–18^ For a practical guide to DNA origami, we recommend the review by Castro *et al.*^19^

### B. Tile-based assemblies

Tile-based methods are common alternatives to DNA origami for designing DNA nanostructures. One such technique, applicable to planar designs, is the single-stranded tile (SST) method, wherein “tiles,” ssDNA molecules comprising four distinct domains, bind to four neighboring tiles [Fig. 1(b)]. First used to create DNA nanotubes with programmable circumferences,^20,21^ this method was later expanded to create a 2D molecular canvas, where each SST acts as a pixel.^22^ Complex planar shapes can be easily designed by using a subset of strands that make up the canvas, analogous to selectively turning on/off pixels to create an image on a screen, see Fig. 1(b). DNA bricks are the 3D extension of this concept,^23^ wherein each DNA “brick” binds to four neighboring bricks to create a 3D canvas. Similarly, a subset of DNA sequences that make up this canvas can be selected to create complex three-dimensional objects, this time analogous to a set of voxels creating a 3D object in space. These techniques are capable of creating structures of similar complexity to DNA origami, without the need for a large scaffold strand. As a result, they present multiple benefits over DNA origami. The most significant benefit of using tile approaches over DNA origami is that, unlike DNA origami, where each DNA structure requires a new set of staples and a scaffold routing scheme, many distinct structures can be formed by using different subsets of the same tiles/bricks. Furthermore, the complexity and size of the possible structures are not limited by the length of the scaffold strand used. In fact, by using a slightly altered brick motif, complex gigadalton 3D structures have been demonstrated,^24^ a feat that, although possible through the combination of DNA origami and natural assembly principles,^25,26^ remains difficult for origami techniques. However, these benefits come at the cost of some flexibility and resolution in the possible designs. Moreover, the yields for megadalton and larger tile structures are significantly lower than those of DNA origami structures.^23,24^ In a broader scope, tile methods need not be limited to these specific approaches and can be generalized for other purposes, such as wireframe structures.^27^

### C. Small kilodalton assemblies

Besides DNA origami and tile assemblies, it is noteworthy that smaller assemblies containing only a few strands have also been shown to have numerous applications. While we include selected examples of applications with these small structures, we primarily focus this review on larger-scale DNA nanostructures, for which shape and geometry are the more useful conceptual frameworks. We direct the readers to the following reviews that have more detailed discussions on the applications of small kilodalton-scale systems.^28–30^

### D. Dynamic structures

Beyond static structural DNA nanotechnology, the functionality of DNA systems has been significantly broadened through methods for creating dynamic structures. Naturally, dynamic functionality necessitates the use of mobile components within a design. Given the drastic difference in the flexibility between single-stranded DNA and double-stranded DNA, this is most commonly achieved in DNA structures by the addition of short (a couple of bases long) ssDNA regions within a design to serve purposes analogous to hinges and ball joints.^31–33^ One can further take advantage of polymer elasticity and employ larger stretches of ssDNA as entropic springs, thereby applying precise forces between components of the structure or between the structure and other molecules.^34–37^ Other types of constrained motion can be achieved through sophisticated design, for instance, by threading a hollow cylinder onto a DNA nanotube and, thereby, confining its motion along one dimension.^31^ Actuation of such devices has been achieved through toehold-mediated strand displacement,^38^ as well as thermal, electric, magnetic, salt-based, pH-based, light-based, and aptamer-based actuation.^39^ We direct the reader to the excellent review by DeLuca *et al.* on the design and methodologies of dynamic DNA structures^39^ and the review by Castro *et al.* on how these systems can be leveraged for molecular-scale precision measurements.^40^

### E. Functionalizations

In addition to the specificity, resolution, and programmability of static and dynamic DNA constructs, the applications made possible by this technology are greatly expanded by the numerous possible chemical functionalizations that can be added to DNA structures. Some of these include fluorescent dyes and quenchers, hydrophobic groups (cholesterol, tocopherol, diacyl lipids, multichain lipids, etc.), electroactive components (methylene blue, ferrocene, porphyrin, pyrene chromophores, etc.), polymers, peptides, and photoreactive compounds (azobenzenes).^41^ These functionalizations lie at the heart of some of the technology's most promising applications: to spatially organize countless biological and synthetic materials and particles with nanometer precision in periodic arrays or anisotropic arbitrarily shaped nanostructures. This makes DNA nanotechnology an area of particular interest in biosensing,^29^ single-molecule studies,^37,42–45^ molecular force sensors,^40,46^ nanophotonics,^47^ drug delivery,^48^ and other fields. Similarly, lipid membrane applications often rely on functionalizations, as we will explain in Sec. IV.

### F. Computational tools and challenges

As the experimental capabilities have progressed, so too have the computational tools developed to facilitate the design of DNA constructs^14,18,26,49^ and to verify their shape and structure using finite element methods^19,50^ and course-grained simulation.^51,52^ These design tools, in conjunction with the robustness of experimental methods, have driven the almost exponential growth that we have seen in the field over the past two decades.^53^ Furthermore, due to the increasingly rapid advancements in artificial DNA synthesis, resulting in higher efficiencies and a drop in cost, what was once the major hurdle for any DNA nanotechnology endeavor is quickly becoming an easily ignored limitation for most research applications. Today, researchers can easily order the needed strands for a DNA origami or SST design over the internet through custom DNA oligo services or take advantage of methods that enable biotechnological mass production of DNA origami.^54^ One critical challenge that still remains, one that is of particular importance for cellular membrane applications, is the stability of DNA nanostructures in cellular environments. For once, the chemical environment within and around a cell is not necessarily ideal, considering for instance, the low cation concentration or low pH that accelerates structure degradation. Besides this, eukaryotic cells are wary of cytoplasmic DNA as it is often a sign of pathogen infection. In response, they have evolved biochemical means of degrading such foreign DNA, such as the Toll pathway.^55^ Scientists have made headway on several methods that can curtail DNA nanostructure degradation in these environments. The issue continues to pose a challenge, though, and it has been discussed in multiple recent reviews.^56–58^

Although DNA nanotechnology remains a nascent field, its scope has expanded beyond what can be covered here. We have omitted several advancements, including curved DNA origami techniques, supramolecular DNA assembly motifs, DNA block copolymers, RNA nanotechnology, and several others. For a comprehensive review of the history of DNA nanotechnology, including all major advances from its inception to 2017, we direct the reader to the excellent review by Seeman and Sleiman.^59^

## III. LIPID MEMBRANES AND CELLULAR MEMBRANES

To understand the physical principles underlying potential applications in which DNA nanotechnology interacts with lipid membranes, it is important to review some key aspects of membrane structural biology and biophysics.

### A. The structure of lipid membranes

The structural backbone of biomembranes is the lipid bilayer. It forms when lipid molecules spontaneously assemble in aqueous solution into thin but highly stable films, whose width is just 4 to 5 nm, but whose lateral extension can easily be three or four orders of magnitude larger (giving them an aspect ratio similar to that of large sheets of paper). This self-assembly occurs because the amphipathic nature of lipids, together with their roughly cylindrical shape, leads them to form a double sheet in which the hydrophobic hydrocarbon chains of the lipid “tails” point to the inside, while the hydrophilic “head groups” are in contact with water. Since no chemical bonds are needed to hold the lipids together, the emergent structure is a two-dimensional fluid, in which lipids can laterally diffuse, with $D=5\;\mu m^{2}/s$ being a fairly typical value for the diffusion constant.^60^ Below a characteristic temperature $T_{m}$, a lipid bilayer enters a more ordered “gel-phase,” in which the area per lipid is smaller, chain order is higher (we might even see a collective lipid tilt), and the membrane becomes stiffer;^61–65^ also, the diffusion constant drops by one or two orders of magnitude.^62^ Gel phases rarely arise in a biological context, though, with the notable exception being the stratum corneum.^66^

The contrast between a strongly hydrophobic interior and a hydrophilic surface region is crucial for the way in which lipid bilayers interact with other molecules. In biology, they solubilize a host of other amphipathic molecules, ranging from small molecules (alcohols, anesthetics, and neurotransmitters) to medium sized peptides up to large proteins that might adsorb onto or insert into the membrane.^67^ The latter is typically driven by hydrophobic anchors (such as fatty acid chains or hydrophobic loops in proteins) that plumb into the membrane or by the fact that the surface of an integral membrane protein's transmembrane region is formed by hydrophobic amino acids. We will see numerous examples for this in Sec. IV.

Lipid molecules come in many different types, which can equip membranes with numerous biophysical properties.^68,69^ For instance, just within the class of glycerophospholipids, we find different tail lengths (with longer tails making membranes thicker and more rigid), different amounts of double bonds (which increase disorder and, hence, membrane fluidity), different hydrogen bonding capabilities in the head group region (which affect lipid packing), and different head group charges (which influence protein binding and membrane potential). If that were not enough, many different classes of lipid exist (such as phospholipids, sphingolipids, plasmalogens, triacylglycerols, and sterols), so that the combinatorial multiplicity easily leads to hundreds of different lipid types—a lavish diversity whose biological function is still largely unknown.^68–71^

Taken together, we recognize a biomembrane as a highly complex, strongly anisotropic, and fully self-assembled molecular composite. It forms an excellent seal between the two half-spaces it separates, but due to its noncovalent nature, it permits a broad array of transport mechanisms. For instance, small neutral molecules (e.g., oxygen, water, and steroids) may easily diffuse through a bilayer without perturbing it, whereas the passage of small ions is almost completely blocked due to the high electrostatic Born energy that a localized charge experiences in a membrane's low-dielectric interior.^72^ Medium-sized molecules (up to a few hundred Dalton) can also diffuse through the bilayer, and their permeability correlates well with their water–octanol partitioning coefficient (a common proxy for water-membrane partitioning),^73^ but even some charged molecules can cross with surprising ease (such as many cell penetrating peptides), by creating localized distortions of the bilayer structure.^74^ Even larger objects (tens of times larger than the membrane width) typically enter via large-scale “engulfment” processes, which can be either passively driven by particle-membrane adhesion^75,76^ or by active cellular processes such as endocytosis.^77^

### B. The mechanics of lipid membranes

On scales just a few times larger than a bilayer's width, lipid membranes can be described with astonishing accuracy as elastic continua. Many DNA origami structures fall into this length scale range, and hence, membrane elasticity offers a powerful predictive framework for assessing the ways in which such structures interact with membranes. Let us here revisit some of the basics.

Imposing a (positive) area strain *u* results in a mechanical membrane tension $\sigma =K_{A}u$. For a wide range of different lipids, the area expansion modulus *K_A_* for simple fluid membranes is remarkably similar, about 250 mN/m.^78^ This value is large, in the sense that even percent-level strains may incur energies much larger than other energy scales in the problem, and so it is often legitimate to model lipid bilayers as inextensible.

For thin sheets—like membranes or paper—the more important mode of deformation is bending. The associated bending modulus *κ* can be estimated using simple continuum models, which show that $\kappa \approx K_{A}d^{2}/n_{\kappa }$, where *d* is the membrane thickness and $n_{\kappa }$ is a number that depends on details of the model (e.g., $n_{\kappa }=24$ for the polymer brush model,^78^ $n_{\kappa }=36$ for two incompressible sliding elastics,^79^ and $n_{\kappa }=48$ for the uncoupled fluid sheets model^80^). Substituting d≈4nm yields bending rigidities in the range of $20-40{\;k}_{B}T$, in agreement with values measured over the past few decades.^78,81–85^ Addition of sterols often stiffens membranes,^86–88^ but not for every type of lipid.^89,90^ Also, recall that gel phase membranes are much stiffer than fluid ones, by about an order of magnitude.^61–65^

A membrane's bending energy is the surface integral over an energy density that is a quadratic in the membrane curvature,^91^

$$E=\int dA\;\left\{\sigma +\frac{1}{2}\kappa {\left(K-K_{0}\right)}^{2}+\bar{\kappa }K_{G}\right\}.$$ (1)

The first term measures the energetic cost of pulling in the additional membrane area from a reservoir against a lateral tension *σ*. It is often just called the “surface tension,” but this terminology is potentially misleading because *σ* typically encodes a boundary condition, not a material property; in particular, its value is not just the oil–water surface tension (on the order of 50 mN/m for small *n*-alkanes^92^) but typically two to three orders of magnitude smaller in resting cellular plasma membranes.^93^ The remaining terms account for curvature elasticity, expressed using two new geometric variables: the total curvature $K=c_{1}+c_{2}$, which is the sum of the two local principal curvatures, and the Gaussian curvature $K_{G}=c_{1}c_{2}$, which is their product.^94^ The first curvature term quadratically penalizes the difference between a membrane's geometric curvature *K* and its spontaneous curvature *K*_0_ (a material property) with a strength given by the bending modulus *κ* we have just discussed. The second curvature term describes the cost of Gaussian curvature ($\bar{\kappa }$ is called the “Gaussian curvature modulus”). However, this term is usually irrelevant because the surface integral over $K_{G}$ depends only on the topology and boundary (i.e., not explicitly on the actual shape).^94^ It is important, though, that membranes—by virtue of being fluid—can change their local Gaussian curvature. This permits initially flat membranes to envelop spherical objects without producing wrinkles, something that thin sheets of fixed local Gaussian curvature cannot do (a perennial problem in the gift wrap industry).

In equilibrium, membranes assume a shape that minimizes the energy in Eq. (1), but finding that shape requires solving a very complicated fourth order partial nonlinear differential equation,^95,96^ a task that has been systematically taken up only in numerical studies of the axisymmetric case.^97^ The problem dramatically simplifies if the membrane only weakly deviates from an approximately flat reference state and can be described by a function *h*(*x*, *y*) above some reference plane (usually pictured “horizontal”). We then have K≈Δh for the curvature and $dA\approx [1+\frac{1}{2}{\left(\nabla h\right)}^{2}]dx\;dy$ for the area element. A subsequent functional variation then leads to a fourth order partial linear differential equation for the shape,

κ ΔΔh−σ Δh=0, (2)

which is much easier to solve and has indeed been very widely studied. The boundary conditions for Eq. (2) typically require the position and slope to be continuous at the circumference of the region over which one wishes to find the shape.

To make objects adhere to membranes, Eq. (1) must be amended by an adhesion energy. From an individual anchor point of view, we then need to know how much the free energy is lowered per insertion event, but unfortunately, this value is often not known very well. Take cholesterol as an example, the most commonly employed anchor for DNA nanostructures (also see Sec. IV): its water–octanol partitioning coefficient has been experimentally determined as $\;{\text{log}}_{10}(P_{w/o})=3.7$.^98^ From this, we can estimate the membrane binding free energy per anchor to be about $8k_{B}T$. But cholesterol anchoring has also been studied experimentally, and a much larger binding free energy of about $18.5\;k_{B}T$ has been reported (for cholesterol extraction out of a liquid disordered phase).^99^ While a direct measurement appears preferable to a value inferred from partitioning data, the large anchoring strength appears to conflict with observations by Khmelinskaia *et al.*, who found that a single such anchor is not sufficient to ensure binding of flat origami plates, while two seem to suffice, but even then their placement still matters.^100^ For our order-of-magnitude estimates below, we will compromise at $13\;k_{B}T$.

Adhesion to larger objects is better described by an adhesion energy density *w*, whose contact area integral yields the overall binding (free) energy. This is especially instructive when dealing with curved substrates, for which adhesion must also supply the cost of bending. The condition that the adhesion energy density exceeds the bending energy density, $w\geq \frac{1}{2}\kappa K^{2}$ (assuming for simplicity $K_{0}=0$), leads to an upper bound for the curvature that the membrane is willing to follow, $K\leq \sqrt{2w/\kappa }$, and explains why it is difficult to wrap around “sharp corners.”

As an example, a tensionless membrane wrapping around a sphere of radius *R* requires a bending energy of $E_{\text{bend}}=4\pi R^{2}\times \frac{1}{2}\kappa {\left(1/R+1/R\right)}^{2}=8\pi \kappa$, independent of *R*. If $\kappa =25\;k_{B}T$, we need about 50 cholesterol anchors. Since these cannot be placed at an arbitrarily large area density, there is an upper bound to *w* and, hence, a lower bound to the size of spheres that can be wrapped. From $8\pi \kappa \leq 4\pi R^{2}w$, we get $R\geq \sqrt{2\kappa /w}$. For instance, if we place cholesterol anchors at a medium area density, one per 14 nm×6 nm patch (an area equivalent to about 130 lipids in a membrane leaflet), we get $w\approx 0.15\;k_{B}T/{\text{nm}}^{2}$, and thus, R≳18 nm. Spheres smaller than that cannot be enveloped by this adhesion-driven process, unless one places the cholesterol anchors more densely or exchanges them for stronger anchors. For spheres, the minimum *w* needed for adhesion to tensionless membranes is the same as that needed for full wrapping, but in the presence of tension, the second boundary shifts to larger *w*, approximately linearly with tension: $w\geq 2\kappa /R^{2}+\sigma$.^75,76^ The situation becomes more difficult for less symmetric objects, such as ellipsoids^101^ or even more complicated shapes.^102^

We wish to emphasize that the inability of sufficiently small objects to enter cells via passive wrapping events does of course not prevent their uptake by other means. For instance, small objects can enter cooperatively by locally porating the membrane (similar to antimicrobial peptides^103^) or driving budding [like Bin/Amphiphysin/Rvs (BAR) domains^104^] they could be decorated with chemical moieties that assist membrane transport (such as cell penetrating peptides^105,106^) and they could simply exploit active cellular uptake processes (such as endocytosis^77^). With that being said, small and delicate DNA architectures (e.g., a small tetrahedral wireframe) are more vulnerable against degradation by endonucleases than larger and more compact ones,^56^ and care must be taken that uptake of a small labeled structure indeed signifies uptake of that structure and not merely of the label after the structure got digested.^107^

Coming back to membrane-substrate adhesion, an even more interesting situation occurs when a membrane partially adheres to a substrate with some adhesion energy *w* per unit area, and the point of detachment is free to adjust. In that case, a new boundary condition on the contact curvature arises,^108,109^

$$K_{⊥}-{\underline{K}}_{⊥}=K_{\text{cc}}=\sqrt{2w/\kappa }.$$ (3)

It states that the membrane curvature $K_{⊥}$ and the substrate curvature ${\underline{K}}_{⊥}$, both measured in the direction perpendicular to the contact line, differ by an amount that is again determined by the characteristic adhesion/bending balance that we have encountered above, except that Eq. (3) holds even for nonzero spontaneous curvature *K*_0_ or tension *σ*. Slightly more complicated conditions exist for the adhesion between two membranes.^109^

**FIG. 2.**
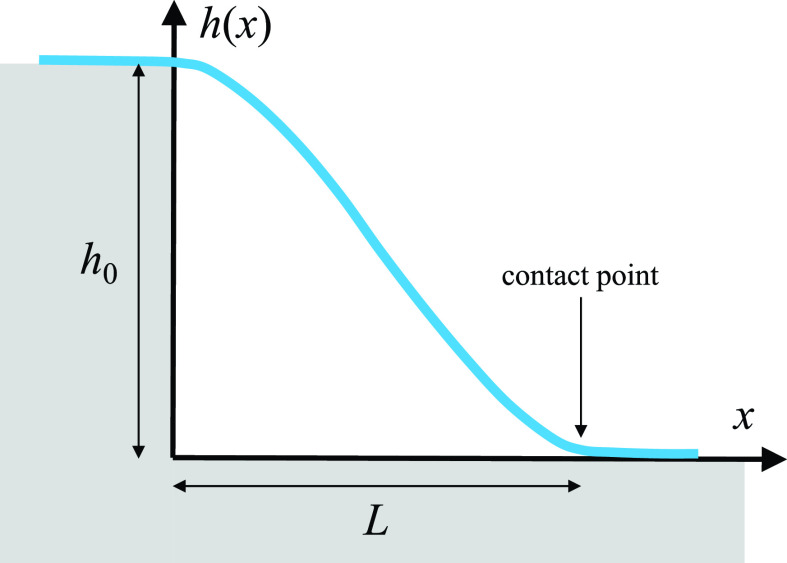
Illustration of a membrane that spans across a step-edge of height *h*_0_, reconnecting with the substrate a distance *L* past the edge.

Let us illustrate all this with two simple toy examples. Assume a tensionless membrane of rigidity *κ* covers a step edge of height *h*_0_, as illustrated in Fig. 2. It has an adhesion energy *w* toward the substrate and reattaches to it a distance *L* away from the edge. How large is *L*? In the absence of tension, and for the one-dimensional case considered here, the shape equation (2) becomes $h^{\prime \prime \prime \prime }(x)=0$, which has the general solution $h(x)=a_{0}+a_{1}x+a_{2}x^{2}+a_{3}x^{3}$. The obvious boundary conditions $h(0)=h_{0},\;h\prime (0)=0$, *h*(*L*) = 0, and h′(0)=0 determine the integration constants $\left\{a_{0},a_{1},a_{2},a_{3}\right\}$ and lead to the solution $h(x)/h_{0}=1-3{\left(x/L\right)}^{2}+2{\left(x/L\right)}^{3}$. The value of *L* is determined by the contact curvature condition (3), which reads $h\prime \prime (L)=K_{\text{cc}}$ and leads to $L=\sqrt{6h_{0}/K_{\text{cc}}}$. For instance, if the jump is $h_{0}=10\;\text{nm}$, the bending rigidity is $\kappa =25\;k_{B}T$, and we again take the adhesion energy density $w=0.15\;k_{B}T/nm^{2}$, we find a contact curvature radius of $K_{\text{cc}}^{-1}\approx 9\;\text{nm}$, and from this, L≈23 nm. This is remarkably large and shows that it might take a sizable adhesion energy to make membranes closely follow corrugated substrates.

**FIG. 3.**
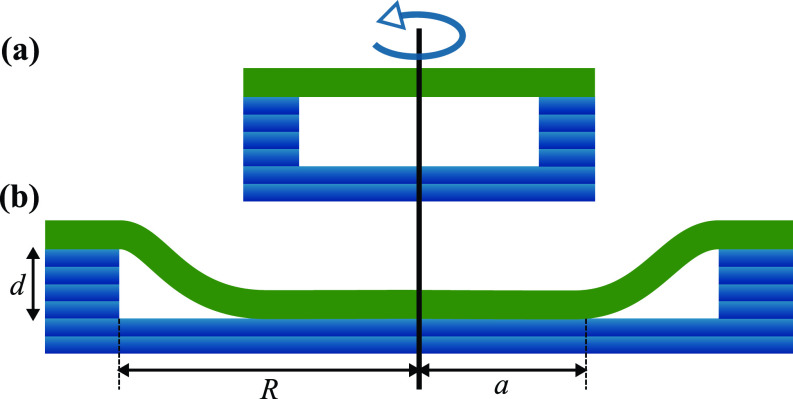
Illustration of tensionless membranes spanning circular depressions, of depth *d*, in DNA structures. (a) Case where the membrane is unable to invade the hole. (b) A similar case with a sufficiently large depression radius (*R*) for the membrane to come in contact with the bottom at a distance (*a*) from the center. Note: this illustration does not indicate a valid method for creating circular indentations in DNA structures and is only intended to give a sense of scale.

A related (but physically richer) scenario is a tensionless membrane covering a circular depression of depth *d* and radius *R*. Will such a membrane just span the hole like the head of a drum or actually “invade” it—see Fig. 3. If the latter, what fraction of its floor will the membrane touch? In cylindrical coordinates, the shape equation ΔΔh(r)=0 has the general (axisymmetric) solution $h(r)=b_{0}+b_{1}\;\text{log}\;(r/R)+b_{2}r^{2}+b_{3}r^{2}\;\text{log}\;(r/R)$, and applying the obvious boundary conditions (assuming that the membrane touches the floor for r≤a) yields the equilibrium shape *h*(*r*), parametrized by *a*. Things are more interesting now, as one finds a discontinuous phase transition between a spanning and an invading state—an interesting exercise that we leave to the reader.

One might worry that these examples are too simple; in fact, it maybe not even realizable: we cannot engineer perfectly crisp step edges with DNA origami, let alone perfectly round holes. However, the repeated general lesson that membranes simply cannot follow surface corrugations smaller than a characteristic length $∼\sqrt{\kappa /w}$ means that we do not, in fact, need to worry about surface roughness below that scale. In other words, if we build a 3d structure from DNA bricks, Minecraft style, then (to a good approximation) we may ignore “voxelation noise” if it remains below the characteristic bending-adhesion length.

### C. Lipid bilayers vs biomembranes

The lipid bilayer is the core structural element of every biomembrane, but the makeup of real biomembranes is significantly more elaborate. Their complexity derives from a number of aspects; chief among them are

**FIG. 4.**
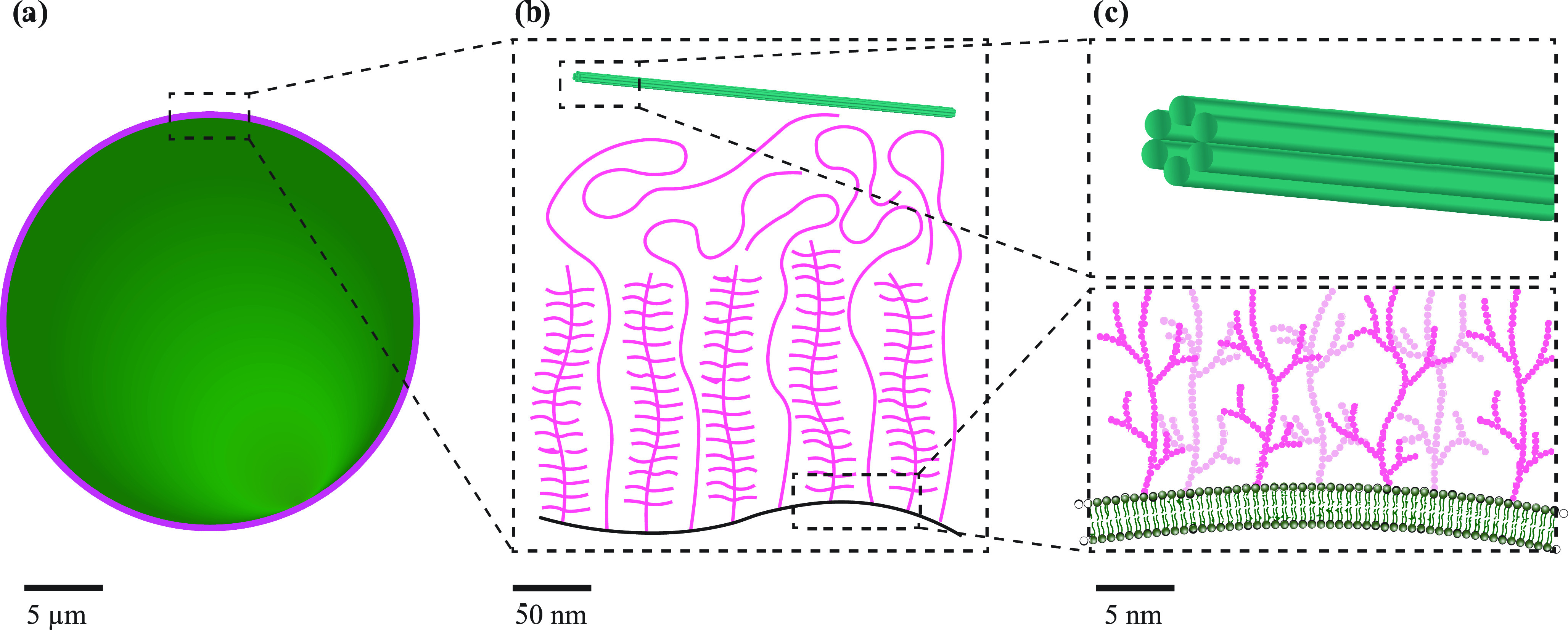
A size comparison between (a) a cell with its membrane-bound glycocalyx, (b) the glycocalyx and a six helix bundled DNA origami tube, and (c) zoomed-in detail views of the glycocalyx and the DNA origami tube. Scale bars are marked in each figure. The length and density of a cell's glycocalyx layer can potentially be investigated by using DNA structures as suitably designed rulers because the positions of decorated moieties on DNA nanostructures can be very precisely controlled.

1. Biomembranes are mixtures of hundreds of different lipid types, with the total cellular lipidome exceeding 1000 different lipids.^68,69^
2. These lipids are not evenly distributed: the outside of cellular plasma membranes is laterally inhomogeneous, containing domains with a length scale of several tens of nanometers that are called “lipid rafts.”^110–112^ The word “domain” is misleading, though, since their small size indicates that they cannot simply be phase separated regions but must be more transient entities. For instance, it has been suggested that rafts are critical fluctuations of a system capable of undergoing a liquid-ordered/liquid-disordered ($l_{o}/l_{d}$) phase separation upon further cooling,^113^ with rafts being the precursors of the more highly ordered and somewhat stiffer $l_{o}$ phase. Since certain types of proteins preferentially partition into rafts,^110,114,115^ the lateral lipid inhomogeneity translates to an inhomogeneity in protein content. Likewise, DNA nanostructures might interact differently with $l_{o}$-like or $l_{d}$-like regions on the cell surface or with the proteins and protein-cluster preferentially found in those.
3. Biomembranes are often also asymmetric, with one of the two leaflets containing a significantly different lipid composition from the other one. This was first established in red blood cells^116,117^ and platelets,^118^ but it was soon found that it also applies to the plasma membranes of nucleated cells.^119–121^ Lorent *et al.* have recently given evidence that suggests that this asymmetry is conserved across the entire domain of eukarya.^71^ Asymmetry matters because it generally leads to a spontaneous membrane curvature; but it can also lead to a stress difference across the two leaflets,^122^ which, in turn, can substantially impact a membrane's curvature rigidity.^123^
4. Biomembranes contain a very sizable fraction of proteins, between 30% and 70%, depending on the membrane type.^67^ These come in three kinds: integral membrane proteins, which contain at least one part that completely passes through the membrane (typically one or several alpha helices); peripheral membrane proteins, which remain outside the bilayer but are anchored to it noncovalently (e.g., by a hydrophobic loop in the protein); and lipid-bound proteins, which are also outside the bilayer but are covalently attached to a lipid (one can also think of them as proteins with lipid anchors). Hence, biomembranes are composites, whose material properties are nontrivially affected by the proteins; at high density (such as in the inner mitochondrial membrane, which is very densely packed with the proteins that form the electron transport chain), they can even obstruct access to the lipid bilayer.
  However, DNA nanostructures could also be designed to specifically interact not with the lipid bilayer but with the proteins bound to it, for instance, with the goal of organizing them into some specific pattern. Given that ligand-induced receptor dimerization is a well-known mechanism for triggering a signaling cascade inside the cell,^124^ DNA nanostructures could be tailored to probe, induce, or interfere with signal transduction processes.
5. Biomembranes may be attached to different types of polymeric networks, and this is especially true for the plasma membrane. For example, endothelial cells are typically surrounded by a glycocalyx, a network comprising proteoglycans and glycoproteins, anchored to the luminal leaflet via glycolipids.^125^ Figure 4 illustrates that the extent of the glycocalyx, while possibly small compared to the size of the cell, may well be large compared to the thickness of the membrane. One may, hence, wonder whether it actually dominates the biomembrane's elastic response. This depends on the scale that one wishes to probe, however. The polymeric network of the glycocalyx has a mesh size estimated to be on the order of 100 nm,^126,127^ and so deformations of the cell membrane and its attached glycocalyx on much larger scales need to take the network into account. But membrane deformation on smaller scales does not require the glycocalyx to deform, and so it can be ignored.

On the cytoplasmic side of the membrane, we have a different polymer network, the actin cortex. Its mesh size depends on the cell type, but again is typically in the 100 nm range.^128,129^

Besides elastic considerations, another obvious effect of such a network, especially the glycocalyx, is that it may obstruct direct access to the lipid bilayer by more bulky objects, such as larger origami structures—either by plain steric hindrance or because of adhesion to the network (like in gel electrophoresis). Since the glycocalyx has a net negative charge,^125^ electrostatic binding may be less of a problem for DNA origami, except in the presence of multivalent cations that could act as linkers.

## IV. PROGRAMMED DELIVERY OF DNA NANOSTRUCTURES

If we wish to use DNA nanostructures to interact in a controlled way with biological systems, in particular to deliver these structures onto, into, or through membranes, it is crucial to understand the “code” with which they “talk” to lipid membranes. In the past few years, several strategies for this have been developed, often with the explicit aim of achieving programmed delivery.

### A. DNA nanostructures on outer surfaces of membranes

**FIG. 5.**
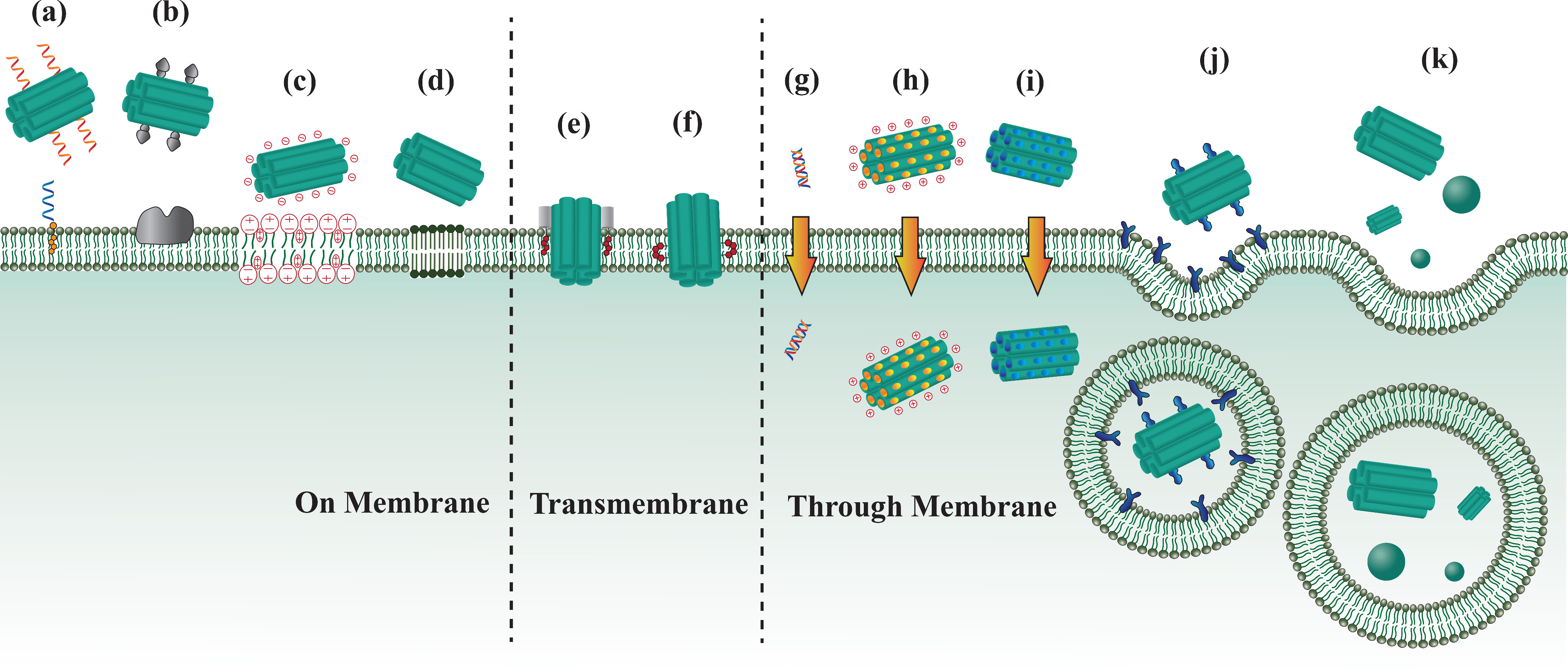
Programed delivery of DNA nanostructures onto, into, and through membranes. Strategies to place DNA nanostructures on the outer surface of lipid membranes by (a) introducing hydrophobic groups, (b) using membrane-associated proteins as anchors, (c) mediating electrostatic interactions, and (d) tuning the phase state of lipid membranes. (e) and (f) Transmembrane DNA nanopores with hydrophobic anchors. (g) Small molecules can pass through the membrane directly. Cellular uptakes can be facilitated by introducing (h) positively charged moieties and (i) cell-penetrating peptides, (j) mediating receptor-mediated endocytosis, and (k) tuning geometric properties of DNA nanostructures.

For the purpose of placing DNA nanostructures onto the surface of lipid membranes, by which we refer to both artificial lipid bilayers and cellular membranes, one method is to dress them with nonpolar groups that function as “anchors”: extensible molecular appendages that lower their free energy by inserting into the lipid bilayer's hydrophobic core, as explained in Sec. III. Among these anchors, cholesterol tags are the most commonly used [Fig. 5(a)].^100,130–142^ Unfortunately, hydrophobically modified DNA nanostructures have a strong tendency to form aggregates in solution because the hydrophobic anchors can also mediate cohesion between the decorated DNA structures.^134,139,140,143^ However, a study by Ohmann *et al.* recently showcased that such aggregation can be mitigated by tuning sequences, length of ssDNA adjacent to cholesterol, and the number and positions of cholesterol molecules.^144^ Another approach for avoiding aggregation when decorating DNA nanostructures with cholesterol is to carry out a two-step process: first, covalently conjugate cholesterol to small stretches of ssDNA and let these amphiphiles insert into the lipid membranes. In a second step, add DNA nanostructures modified with the complementary ssDNA, which can hybridize with its membrane-bound ssDNA complement and thus recruit the DNA nanostructures on the lipid membrane.^139,142^ Besides cholesterol, other hydrophobic groups have been successfully used as anchors, such as porphyrin ethyl phosphorothioate (EP)^145^ and *α*-tocopherol.^146^

An important question that arises with this approach is how the number and nanoscale spatial distribution of hydrophobic anchors affect the membrane attachment. Researchers have noticed such effects previously.^137,147^ In 2016, Khmelinskaia *et al.* performed a more systematic study on this topic. To investigate this, the authors constructed rectangular-shaped 20-helix bundles with 15 potential cholesterol-tetra-ethylene glycol (chol-TEG) anchors that were spatially evenly distributed on the outer surface of the structures.^100^ By systematically altering different combinations of anchors on the DNA nanostructure with varying numbers and spatial configurations, and comparing membrane binding efficiency via fluorescence measurements, they found that two anchors generally sufficed to bind DNA structures to artificial lipid bilayers, which is compatible with our estimate of $13\;k_{B}T$ for the binding strength of a single cholesterol anchor (see Sec. III B). With an increasing number of anchors, the attachment tended to be stronger. This study also demonstrated that anchor position affected membrane accessibility by individual anchors.

Another approach for anchoring DNA nanostructures to lipid membranes is to use membrane-associated proteins [Fig. 5(b)]. An autonomous payload-carrying DNA “nanorobot” was designed and manufactured with an aptamer lock-and-key system.^148^ The unlocking and release of payloads, fluorescently labeled antibodies, was activated by aptamer recognition. Subsequently the freed antibodies bound to specific antigens on the cell membrane, which led to the attachment of the nanorobot to the cell membrane. Six cell lines were tested using nanorobots carrying different aptamers. The number and nanoscale spatial distribution of membrane-associated proteins have also been shown to be important.^149^ An increase in the number of ligands and their separation on the DNA nanostructures generally enhance their binding ability to receptors on the cell membrane. Ligands such as epidermal growth factor (EGF)^150^ and synthetic cell adhesion peptide Arg-Gly-Asp-Ser (RGDS),^151^ have also been reported to bind cell membrane-bound receptors.

DNA nanostructures can also be placed on supported lipid bilayers (SLBs) by mediating electrostatic interactions between them [Fig. 5(c)]. Negatively charged DNA are electrostatically attracted to polar or positively charged lipid headgroups of SLBs. For noncharged SLBs like zwitterionic lipid layers, the binding of DNA and lipids can be achieved by mediation of divalent cations.^152^ In one study, cross-shaped DNA origami with a blunt end were electrostatically absorbed to zwitterionic lipid bilayer surfaces under the presence of divalent cations.^153^ 2D lateral lattice formation was assisted by lipid membranes with stacking interactions between blunt ends. Similar results were reported by another study, which constructed cholesterol-modified three-point star (3PS) DNA tiles and showed that they bound to supported lipid bilayers, where they further assembled into a hexagonal lattice.^154^ Interestingly, the authors found that the phase state of the lipid bilayer could also modulate the process [Fig. 5(d)]: on fluid bilayers, cholesterol tiles formed organized arrays, while tiles without a cholesterol anchor did not bind; in contrast, tiles with or without a cholesterol anchor bound to gel-phase bilayers and formed organized morphologies. Such a phase tuning method was utilized by Sato *et al.* to facilitate the attachment of cross-shaped DNA origami to the lipid bilayer membrane.^155^ In a phase-separated lipid bilayer membrane consisting of liquid-disordered and solid-ordered phases, DNA nanostructures tended to bind to solid-ordered phases more readily as compared to liquid-disordered phases. The concentration of NaCl was also shown to be important in modulating the process. The authors achieved successful self-assembly of blunt-ended DNA origami on the membrane, opening up new opportunities for DNA-based elements to assemble into higher order functional nanodevices in a lipid-phase and ion-responsive manner.

It is worth noting that both mediating electrostatic interactions and tuning phase state of lipid bilayers have only been applied to artificial lipid bilayers. Considering the complex cellular surface environment, as discussed in Sec. III C, many additional challenges arise when using DNA nanostructures on cellular membranes. More work is clearly needed to address these.

### B. Transmembrane DNA nanostructures

DNA nanostructures have the potential to function as synthetic lipid membrane channels for mimicking biological functions in lipid membranes. To insert functional DNA structures into the membrane, two components are typically needed with one component responsible for adhering structures to the membrane and the other to span structures in the membrane.

Transmembrane fixation can be achieved by designing a “cap”-like component to the DNA nanostructure, which is attached with hydrophobic or other anchors and can help to insert the whole structure into the lipid membrane [Fig. 5(e)]. In this work by Langecker *et al.*, DNA-based synthetic artificial lipid membrane channels were designed.^147^ A barrel-shaped cap was attached to the lipid bilayer through 26 cholesterol moieties, carrying a six helix bundled stem that can penetrate and span the membrane. In another study, Göpfrich constructed a megadalton funnel-shaped DNA origami porin (a beta barrel protein) with 19 cholesterol tags which spontaneously inserted into the lipid membrane.^156^

An alternative strategy to insert structures into lipid membranes is to put anchors, mainly hydrophobic groups, on the outer surface of DNA nanostructures to mask the negatively charged DNA backbones, thereby lowering the energy barrier for membrane insertion and attachment [Fig. 5(f)]. Such anchors like cholesterols,^157^ porphyrins,^158^ alkyl groups,^159^ and ethylated phosphorothioate groups^160^ have been reported.

### C. DNA nanostructures through membranes

Cellular uptake is a biological process that transports substrates outside cell membranes into cells. There are two general pathways, passive cellular uptake and active cellular uptake. Factors that determine whether cellular uptake will take place and which pathway the substrate will take include the physical and chemical properties of the substrate, as well as the particular characteristics of the cell membrane for a given cell type. For in-cell functional DNA nanostructures, overcoming the lipid membrane barrier is one of the major challenges in order to achieve in-cell functionalities. Although highly dependent on specific cases, general delivery strategies have been studied in recent years to facilitate cellular uptake, which will be summarized in this review. Other challenges like endosomal avoidance, escape and subcellular localization are also crucial, but we will not cover these topics here and refer the readers to a detailed review.^213^

#### 1. Passive cellular uptake

Passive cellular uptake relies on diffusion, usually driven by the physical and chemical properties of substrates, concentration difference or electric potential gradient. Small molecules like short DNA duplexes can directly diffuse through the membrane [Fig. 5(g)]. For larger DNA structures, as both DNA backbones and most cell membranes are negatively charged, the repulsive electrostatic forces between them inhibit the diffusion of DNA nanostructures through membranes. However, by introducing positively charged moieties to mediate electrostatic interactions, DNA nanostructures can be delivered through lipid membranes directly [Fig. 5(h)].^161–163^ Xu *et al.* coated DNA origami with cationic human serum albumin protein via electrostatic interaction.^163^ The positive encapsulation was able to not only increase the transfection into HeLa cells by threefold, but also improve the stability of DNA origami under physiological conditions and from Deoxyribonuclease I (DNase I), a specific endonuclease commonly used to breakdown chromatin. A similar strategy was applied to coat dendrons of DNA origami with bovine serum albumin through cysteine-maleimide bond.^162^

The ability for DNA nanostructures to penetrate lipid membranes can be enhanced by using cell-penetrating peptides (CPPs) [Fig. 5(i)]. CPPs typically have an amino acid composition and are able to carry cargoes like peptides, proteins, nucleic acids and other nanoparticles (NPs). It is worth noting that depending on the specific type of CPPs, the pathway of passing through membranes varies. Some CPPs cross the membrane via direct penetration while others utilize energy-dependent active cellular processes. For example, cowpea chlorotic mottle virus capsid proteins were modified to the outer surfaces of rectangular and tubular shaped DNA origami by electrostatic interactions to form capsid protein-origami complexes.^164^ A 13-fold increase in internalization was observed in human HEK293 cell line because of the enhanced penetrating ability provided by the capsid proteins. Qu *et al.* modified their dendrimer-like DNA nanostructures with another commonly used cell-penetrating peptide, transactivator of transcription (TAT) peptide, to deliver immunostimulatory cytosine-phosphate-guanosine (CpG) sequences and activate an immune response.^165^ The TAT-decorated CpG DNA nanostructures were found to have better immuno-stimulating effects in RAW264.7 macrophage-like cells, indicating an increase in cell internalization and cytokines production. A similar work was done by Yan *et al.* where gold nanoparticles (AuNPs)-DNA belts structures were constructed using rolling circle amplification (RCA) and were loaded with CPPs.^166^ Structures with CPPs were shown to more readily be internalized. For further information on CPP types, transduction mechanism, applications, especially nucleic acids like DNA origami applications, and existing challenges, we recommend an extensive review of CPPs for gene therapy by Taylor and Zahid.^106^

#### 2. Active cellular uptake

Compared to passive cellular uptake, energy-dependent active uptake, in which DNA nanostructures with modified ligands trigger specific receptors on the membrane, are generally more efficient. It stimulates cell membranes to engulf substrates and bring them into cells. One example is to facilitate receptor-mediated endocytosis [Fig. 5(j)]. Schaffert *et al.* introduced a variable number of functional transferrin-oligodeoxynucleotide conjugates to rectangular DNA origami.^167^ The KB-3-1 (human epidermoid carcinoma cell line) was used and transferrin-mediated endocytosis was a common pathway. Results showed structures with 1, 16 and 32 transferrin molecules had around 8, 14 and 22-fold higher uptake compared to unmodified structures. Cell types also had a great impact on uptake efficiency, presumably due to differences in cell membrane-bound receptors.^168^ Such effects were found to be more influential than substrate shape.^169^

Geometric properties such as size and shape have also been confirmed to have a significant impact on active cellular uptakes [Fig. 5(k)]. Tetrahedron and rod DNA origami nanostructures (DONs) with small and large sizes were constructed and cellular uptake into multiple human cancer cell lines was investigated.^170^ Scavenger receptors were found to be critical in mediating the endocytosis of DNA structures. Moreover, rod shaped structures were internalized faster than tetrahedrons. Within each shape, larger sizes had higher uptake efficiency. Similar results were shown by examining the uptake efficiency of eleven distinct DNA origami structures in three cell lines.^169^ Large structures with higher compactness were more likely to be internalized compared to elongated, high aspect-ratio ones. However, in another study where rectangular and tubular shapes DNA structures with varying dimensions were developed using the modular DNA brick method, the uptakes of smaller sized structures was faster.^168^ At this stage, few universal agreements have been made in terms of effects of sizes and shapes. More efforts are expected to solve this puzzle, among which molecular simulations are particularly promising. In simulations, structures with controlled size and shape are easy to construct, and their internalization efficiency can be straightforwardly quantified with simulation time. Moreover, simulations are able to visualize the internalization process with high resolution, well below the diffraction limit of fluorescence microscopy techniques. Both molecular simulations and experiments by Ding *et al.* reported that tetrahedral and multihelix bundled DNA nanostructures minimized electrostatic repulsion from lipid membrane with their corners attacking the membrane first, causing regional uneven charge redistribution in the membrane and thus facilitating lipid-raft-/caveolin-mediated endocytosis.^171^ In their simulations, structures typically reached to the membrane within 15 *μ*s. Using ligands coated nanoparticles (NPs), the receptor-mediated endocytosis of spherocylindrical and spherical ligand-coated NPs have also been simulated.^172,173^ Similarly, Huang *et al.* found that there was an optimal size for spherical NPs to facilitate endocytosis.^173^ Since cellular uptake is a relatively long biological process, the computational cost needs to be taken into consideration, which makes coarse-grained simulations more advantageous. With more efforts from simulations and quantitative biophysical research, we foresee the mechanisms of DNA nanostructures cellular uptake to be revealed in the near future.

## V. APPLICATIONS AT THE INTERFACE OF DNA NANOSTRUCTURES AND LIPID MEMBRANES

With an understanding of the principles of DNA nanotechnology, lipid and cellular membranes, and strategies to mediate interactions between them, we now focus on emerging applications at their interface.

### A. Membrane sculpting

Some applications of DNA nanotechnology are based on its ability to deform lipid bilayers by simply anchoring a structure to the surface of a membrane. This capability was first observed in a study of hierarchical superstructures formed from freely diffusing DNA origami constructs anchored to small unilamellar vesicles (SUVs).^139^ This is of particular interest as many cell processes hinge on the presence of precise membrane curvatures. Proteins of the BAR (Bin/Amphiphysin/Rvs) domain superfamily are the most prevalently tasked with inducing local membrane curvature and membrane remodeling over large distances. Their function has been shown to play key roles in endocytosis, regulation of intracellular organelle shape and cell signaling, acting as nucleating platforms for actin polymerization, and others.^178^

Inspired by these proteins and their importance in cellular environments, later studies aimed to more closely replicate this membrane sculpting functionality using DNA nanostructures. One such study used DNA origami constructs with planar faces to replicate PinkBAR domain, a protein that is almost completely flat and that induces planar deformations on cell membranes.^179^ The planar surface of the DNA structures that made contact with the membrane was roughly 50 nm × 40 nm and included nine cholesteryl-TEG modified staples as anchors. The top of the structure was decorated with ten fluorophore modified staples to facilitate fluorescence imaging. The lateral faces of the constructs included ssDNA overhangs, protruding ssDNA molecules, that were designed to promote the oligomerization of multiple structures into a brick-wall like pattern. When the coverage of these DNA superstructure on SUVs reached similar levels to those required by some BAR domains to deform giant unilamellar vesicles (GUVs), a majority of SUV's assumed nonspherical shapes characterized by planar deformations similar to deformations caused by PinkBAR domain.

**FIG. 6.**
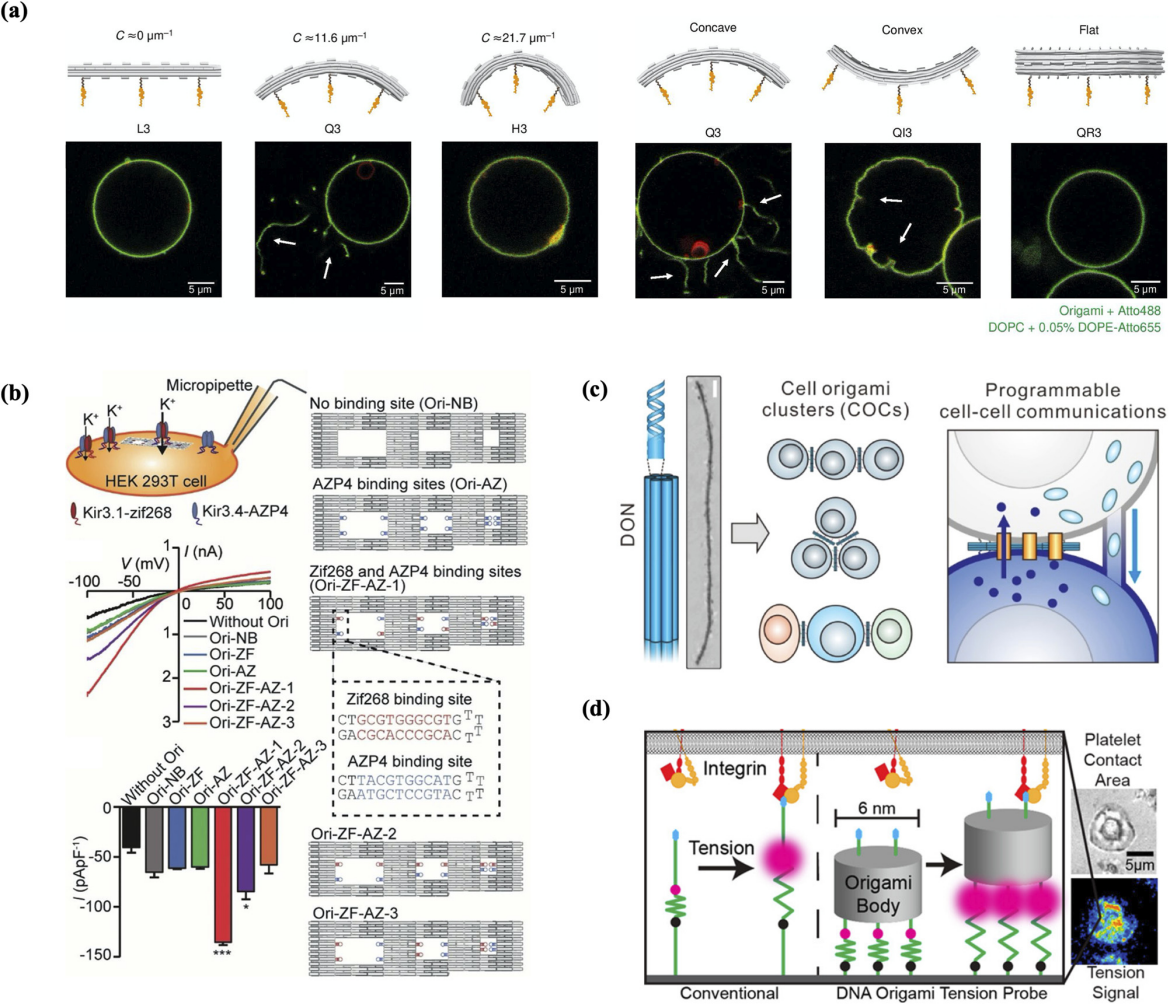
On-membrane DNA nanostructures: (a) curved DNA structures developed to deform lipid membranes. Flat structure L3 has no effect on the GUV. Structure Q3 promotes the tubulation of the membrane, while structure H3 is unable to do so. The concave version of Q3, QI3, caused invaginations in the membrane. Q3 with the anchors on its side, QR3, promotes no tubulation.^174^ Reproduced with permission from Franquelim *et al.*, Nat. Commun. **9**, 1 (2018). Copyright 2018 Authors, licensed under a Creative Commons Attribution (CCBY) License. (b) DNA nanostructure used to promote the assembly of tetrameric Kir3 K^+^ channels. When a heterotetramer binding site configuration is used, a roughly threefold increase in whole-cell K^+^ currents is observed when compared to DNA constructs with no binding sites.^175^ Reproduced with permission from Kurakawa *et al.*, Angew. Chem., Int. Ed. **57**, 10 (2018). Copyright 2018 Authors, licensed under a Creative Commons Attribution (CCBY) License. (c) DNA origami nanostructures (DONs) assist homotypic and heterotypic cell origami cluster (COC) formation in linear and closed-ring topologies and programable cell–cell communications.^176^ Reprinted with permission from Ge *et al.*, J. Am. Chem. Soc. **142**, 8800 (2020). Copyright 2020 American Chemical Society. (d) Multivalent DNA origami tension probes with a tailorable number of hairpin molecules can report cellular traction forces by human blood platelets.^177^ Reprinted with permission from Dutta *et al.*, Nano Lett. **18**, 4803 (2018). Copyright 2018 American Chemical Society.

Although this was a demonstration of DNA constructs' ability to shape membranes, replication of BAR domain's ability to form curved deformations was not achieved until 2018 when Franquelim *et al.* designed DNA origami nanostructures that mimicked the molding properties of BAR domains with different degrees of curvature.^174^ They designed two curved DNA origami structures, one resembling a quarter circle, Q3, ($C\approx 11.6\;\mu m^{-1}$) and one resembling a half circle, H3, ($C\approx 21.7\;\mu m^{-1}$). It is noteworthy that these DNA origami mimics were five times larger in size than their biological counterparts. Nevertheless, when attached to GUV's and upon hyperosmotic shock, they demonstrated that their moderately curved structure was able to trigger tubulation of the membrane, similar to tubulation caused by some curved BAR domain proteins, see Fig. 6(a). The concentration needed to trigger tubulation was lowered when additional cholesteryl moieties were added. This is easily explained by the increase in binding energy density as explained previously. The concentration needed was also lowered when lateral oligomerization of the constructs was promoted through complementary ssDNA overhangs. When TEG-cholesteryl attachments were instead added to the opposite face (thereby creating a convex membrane–binding interface), invagination-type deformations resembling deformations caused by convex BAR domains were formed. For the more highly curved structure the adhesion free energy provided by the TEG-cholesteryl anchors was not enough to overcome the energetic cost of bending the membrane into tubules of smaller radius, and thus larger curvature, even when the number of cholesterol anchors were increased. This study was able to pinpoint three main requirements for the induction of tubular membrane deformations: curvature, membrane affinity, and surface density. More broadly, it demonstrated DNA nanotechnology's ability to mimic a broad range of biological functions. In a more recent study, the same group developed straight origami DNA filaments that could reversibly deform GUVs.^180^ DNA clathrin-mimics were also shown to cause deformations similar to clathrin-coated pits^181^ and DNA nanosprings to cause GUV tubulation.^182^ It has also been shown that DNA nanopores (discussed in a later section) cluster and remodel membranes into protrusions and can facilitate the formation of ultrathin lipid tubes.^183^ This ability to model membranes has not been demonstrated by biological nanopores and could be the basis of novel synthetic biology applications.

Although DNA BAR domain mimics have yet to be tested on live cell membranes, these studies demonstrate DNA nanotechnology's ability to mimic membrane-bound protein functionality while elucidating physical and chemical underpinnings of biological processes. Still there remain a number of avenues by which these mimics can be advanced that could hold promising applications in cell biology. A function that has yet to be demonstrated by DNA bar domain mimics is their curvature sensing capability. It has been shown that the difference between a BAR domain acting as a curvature sensor vs a curvature inducer is binding affinity.^178^ This is yet another simple consequence of the energetic cost of membrane bending: a curved structure (without the necessary binding affinity to bend the membrane) will attach more readily to portions of a membrane that most closely matches its own curvature. The binding affinity of a DNA nanostructure can be, straightforwardly, tuned by changing the anchor density, so this would be a natural extension to what has already been shown. Other functions include BAR domain's ability to alter membrane liquid properties, promote scission, as well as their responsiveness to physical parameters such as protein surface density and membrane tension and shape. These capabilities could have wide-ranging applications.

### B. Multicell self-assembly

Programmed cell–cell adhesion by embedding DNA nanostructures on the surface of cell membranes offers new opportunities in investigating intercellular communications, tissue morphogenesis and organ development. Functioning as bridges, DNA nanoplatforms can enhance the ability to construct cell clusters with high precision, controlled configurations and inherent reversibility. Todhunter *et al.* created a modular DNA-based method for controlling 3D microtissue structure that they called DNA Programmed Assembly of Cells (DPAC).^184^ In this technique, reductive amination was used to covalently link amine-decorated DNA with aldehyde-coated glass slides. Cells that had been decorated with complementary lipid-modified oligonucleotides were introduced, and in a multistep manner they demonstrated the construction of an extracellular matrix-embedded three-dimensional multicellular organization with controlled size, shape, composition and spatial heterogeneity. The assembly can be reversed using DNase.

In another study, DNA nanoplatforms, which consisted of 34 dsDNA helices and contained 34 ssDNA overhangs, were attached to cell membranes through hybridization between the ssDNA overhangs and cholesterol-conjugated oligonucleotides that were incorporated into the plasma membrane.^142^ In conjunction with binding bridge oligos, intermediate 60-base oligonucleotides, the nanoplatform can serve as a membrane-bound breadboard (MBB) capable of programming cellular assembly. The MBB attachment and detachment to the cell membranes were made specific, controllable and reversible through other functional oligos, such as bridge fortifier oligos that enhanced the attachment, binding inhibitor oligos that prevented the attachment, and detachment oligos that detached DNA origami and cell membranes through strand displacement. Higher-order assemblies of MBBs on cell membranes enabled programmable cell–cell adhesion between homotypic and heterotypic cells.

In a more recent study, DON-based biomimetic membrane channels were applied to program spatial arrangement of homotypic and heterotypic cell origami clusters (COCs) [Fig. 6(c)].^176^ Thiolated ssDNA molecules were inserted into Jurkat cell membranes as anchors. The ssDNA decorated DONs were able to assemble cells in linear and closed-ring topologies. Moreover, three cell–cell communication methods, including gap junctions, tunneling nanotubes and immune/tumor cell interactions, were successfully induced and tuned using artificial COCs. They further utilized the COC system to demonstrate controllable T-cell immunoresponses, which were triggered by interactions between receptors on T cells and antigens on tumor cells. Immunoresponses were significantly enhanced and tumor cell death rates were positively related to the ratio of T cells to tumor cells in COCs. Such DNA-based platforms with controllable size, shape and well-defined positioning of diverse modifications will greatly extend the ability in multicell self-assembly and programmable cell–cell communications.

### C. Spatial arrangement and organization of ligands/proteins

By leveraging its ability to precisely place molecules, DNA nanotechnology can elucidate how the number, spacing, and orientation of ligands and proteins can alter their biological functionality.

Researches have been able to study the role of multivalent ligand spatial arrangement on membrane-bound receptors through ligand presentation studies. In the previously cited work by Shaw *et al.*, they developed DNA “nanocalipers” as the first demonstration of direct regulation of receptor function by nanoscale distribution of ligands.^149^ These nanocalipers were modified with ephrin ligands at precise locations and separations. The nanocalipers were then used to “present” the ligands to cells. They demonstrated that placing two ephrin-A5 ligands on nanocalipers triggered EphA2 receptor activation on human breast cancer cells more efficiently than when just one ligand was added. Moreover, the spacing between ligands affected the level of activation. Specifically, nanocalipers with ligand spacing of 42.9 nm showed more EphA2 activation than 101.1 nm spacing. This work demonstrated the ability of DNA nanotechnology to probe the effects of ligand distribution on the cell membrane.

In a more recent study, Huang *et al.* developed a technique for the fabrication of biomimetic DNA origami nanoarrays that permit the study of multivalent ligand–receptor molecule interactions and its effects on cell spreading.^185^ They accomplished this by organizing DNA nanostructures modified with integrin and epidermal growth factor (EGF) on nanopatterned surfaces. As a demonstration of the capabilities of such a system, they showed a positive cooperative behavior of integrin and EGF ligands in human cutaneous melanoma cell spreading with single-molecule control and nanoscale spatial resolution. In a more recent study by Hawkes *et al.*, the system was extended to study the significance of ligand number, spacing, and multivalency in cardiomyocyte adhesion, spreading, and maturation. They demonstrated a wide range of variation in clustering behavior of different receptor types motivating the further study of nanoscale receptor organization.^186^ A similar strategy was used by Veneziano *et al.* to enhance the efficacy of a HIV clinical vaccine.^187^ Their results showed that increasing the number of immunogens and maximizing their spacing enhanced cell activation. These studies demonstrate the potential of such DNA systems in these applications. There have been numerous studies in this field, for a more in depth discussion we direct the readers to the following excellent review articles.^188–190^

DNA nanotechnology can also be used to recapitulate membrane-like systems for investigating and controlling biological processes. Using a relatively simple DNA hybridization motif, where two proteins were conjugated with ssDNA molecules with complementary regions, Raschle *et al*. managed to drastically increase the yield of protein dimers and trimers on lipid bilayers nanodiscs (nanoscale disc-shaped lipid bilayers constrained by proteins).^191^ Additional examples of DNA-based membrane-manipulation tools include DNA-encircled bilayers (DEBs)^192^ and DNA-corralled nanodiscs.^193^

Controlling biological processes through the precise placement of proteins, Xu *et al.* developed a DNA origami platform composed of a DNA ring with a predetermined number of outward facing SNAREs (soluble N-ethylmaleimide-sensitive fusion protein attachment protein receptors). The DNA origami ring templated uniformly sized SUVs where lipid-conjugated oligonucleotides acted as tethers allowing the circumvention of the rate-limiting docking step that natural SNARE merging events are subject to. Through the observation of individual SUV-supported lipid bilayers merge events with different numbers of SNARE attachments, they were able to confirm that one to two pairs of SNAREs are sufficient to drive membrane fusion.^194^ In another application of DNA nanotechnology, two size-controlled liposomes were confined within DNA rings at precise separations to quantitatively study the lipid transfer between them caused by synaptotagmin-like mitochondrial lipid-binding protein (SMP) domain of extended synaptotagmin 1 (E-Syt1).^195^ They demonstrated that SMP domain could transfer lipids between bilayers at distances that were larger than the SMP domain itself. This approach could be generalized to study other membrane–membrane interactions and showcases DNA nanotechnology's ability for quantitative studies.

The first application of DNA nanotechnology's ability to template proteins in living cells was achieved in 2018 by Kurokawa *et al.* In this study, they created a DNA origami structure to control the assembly of tetrameric Kir3 K^+^ channels.^175^ Kir3 channels are a subfamily of inwardly rectifying potassium channels, channels whose allowed inward flux is much greater than its outward flux. While individual Kir3.1 and Kir3.4, protein members of the Kir3 family, are unable to produce significant transmembrane currents, a heterotetramer assembly, where pairs of Kir3.1 and Kir3.4 are placed at diagonal positions of a square, is thought to be necessary for effective channel activation. Using a planar DNA structure, with three cavities each addressable with the desired binding sites, this study was able to scaffold Kir3.1 and Kir3.4 subunits with different arrangements. When testing whole-cell K^+^ currents in HEK293T cells that contained the DNA origami structures with adapters in the heterotetrameric configuration, they saw nearly three times the current when compared to cells with bare DNA structures [Fig. 6(b)]. This study thereby successfully verified DNA origami's ability to template protein subunits to promote the formation of functional heterotetrameric Kir3 K^+^ channels in live cells. Moreover, as the first *in vitro* demonstration of spatial protein organization, this serves as a demonstration of the efficacy of DNA systems as bottom-up synthetic biology tools for elucidating the chemical and physical properties of multiprotein complexes. It is of particular interest to see how such systems may evolve beyond investigative roles into *de novo* methods for regulating and overcoming limits of natural protein systems.

### D. Biomechanical sensing

DNA-based extracellular biomechanical sensing is another emerging on-membrane DNA nanostructure application. Membrane mechanobiology is an increasingly important area of study due to the importance of mechanical stimuli in many membrane-associated biological processes, such as tissue morphogenesis, cell signaling and differentiation. The ability to quantitatively measure mechanical forces, especially with a high signal-to-noise ratio and sensitivity to the level of fN and pN, has been hugely enhanced by the emergence of modular DNA nanotechnology.^36,197,198^ Decorated with a tailorable number of tension-reporting hairpin molecules that contained a fluorophore and quencher pair, multivalent DNA origami probes demonstrated the ability to increase force-responsive thresholds [Fig. 6(d)],^177^ which is particularly important in situations when the magnitude of the force is unclear. In one example, probes were attached to gold nanoparticle (AuNP)-coated glass slides through thiol-Au binding and to cell membranes by integrin and adhesive peptide (cRGDfk). They successfully mapped cellular traction forces by human blood platelets during initial adhesion and activation.

However, most of these DNA-based biomechanical sensors are utilized in the artificial lipid bilayer environment. There are still challenges in moving toward biological cell membranes due to difficulties in maintaining DNA nanostructure integrity and stability in complex cellular environments and signal detection. With efforts continuously being made, especially on innovative strategies for sensor-membrane attachment, we foresee more progress in *in vitro* and *in vivo* extracellular biomechanical sensing.

### E. Synthetic DNA nanopores

**FIG. 7.**
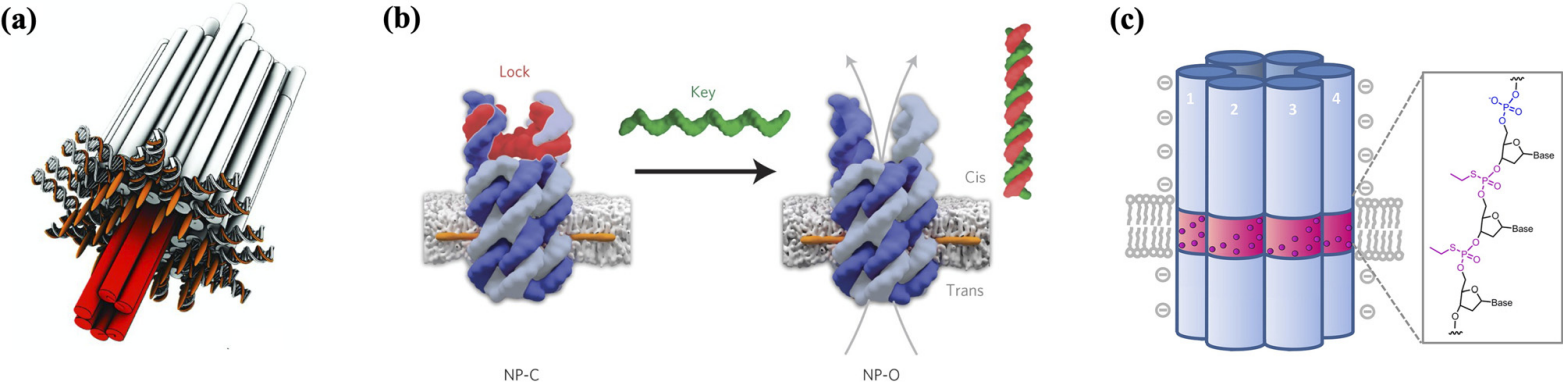
Transmembrane DNA nanostructures. (a) Synthetic DNA-based channels consist of a stem that has membrane penetration and span functions and a barrel-shaped cap that adheres to the membrane using cholesterol moieties.^147^ Reproduced with permission from Langecker *et al.*, Science **338**, 932 (2012). Copyright 2012 AAAS. (b) A transmembrane synthetic DNA-based channel controls the transport of the molecular cargo using a sequence-specific gate that regulates the open and closed states of the channel. Reproduced with permission from Burns *et al.*, Nat. Nanotechnol. **11**, 152 (2016).^196^ Copyright 2016 Springer Nature. (c) A membrane-spanning DNA nanopore that contains a hydrophobic belt made up of ethyl phosphorothioate (EP) groups triggers cytotoxic activity.^159^ Reprinted with permission from Burns *et al.*, Nano Lett. **13**, 2351. Copyright 2013 American Chemical Society.

DNA nanostructures that embed and span membranes can act as synthetic membrane channels or nanopores. These nanopores have demonstrated huge potential in mimicking biological membrane channels as well as other applications such as DNA sequencing and functioning as new agents in therapeutics. One of the earliest DNA nanopores was constructed with a six helix penetrating needle and a base with cholesterol that aided the penetration and adherence to SUVs and GUVs [Fig. 7(a)].^147^ The conductance ability was showcased by a stepwise increase in ionic current after the incorporation of nanopores. Conformation-dependent current gating properties often possessed by natural ion channels were also replicated. Mutated channels with a single-stranded oligomer protruding from the structures showed more pronounced gating compared to wild type channels. Based on temporarily blocking nanopores and then causing ion current change, the unfolding of hairpin and guanine quadruplex through central cores were performed. Such DNA based single-molecule biosensors with custom sizes open up new possibilities for DNA sequencing and precise chemical conjugation.

Further work has seen the simplification of DNA nanopores composed of significantly fewer staple strands and chemical modifications by the Howorka group.^158^ Only two tetraphenylporphyrin tags were conjugated to the outer surface of the six-helix nanopore with 6.5 nm in diameter and 14 nm in height. The nanopore demonstrated the ability to be stably inserted into lipid membranes and maintain integrity. A similar sized DNA nanopore modified with alkyl-phosphorothioates and ethyl moieties was able to perform ion conductance and had gating properties similar to natural ion channels.^159^ Transport of folded proteins like trypsin and enhanced green fluorescent protein (EGFP) through cholesterol-anchored DNA nanopores was also explored by the same group using massively parallel single-channel readout and electrical recordings, revealing the 20-fold slower protein diffusion speed compared to electric field-driven transport.^199^

Other DNA nanopores include a large 9 nm wide DNA nanopore which was created for the size-selective translocation of macromolecules in GUVs and SUVs.^200^ Importantly, a locking mechanism was applied to lock DNA flaps by staple strands so that hydrophobic lipid moieties would be shielded from the aqueous environment and therefore limit hydrophobicity-driven aggregation. DNA flaps can be opened by strand displacement under the presence of complementary ssDNA, which would expose lipid moieties and insert the nanopore into lipid membranes. Instead of using DNA as the main material, ring-shaped DNA scaffolds can direct the assembly of peptides into nanopores with various sizes and uniform conductance.^201^ DNA has also been used to template alpha-hemolysin monomers to create protein pores with designed diameters and conductances.^202^ Another important study brought the application of DNA-based nanopore to specific, controllable and selective transport of molecules [Fig. 7(b)].^196^ Locking mechanisms mediated by strand displacement and ligand activation were able to control the state of the pore (closed or open), leading to selective transport of molecule cargos and flux differences before and after triggered channel opening. In one of the *in vitro* examples, by inserting into cervical cancer cell membranes, ethyl phosphorothioate-coated six helix DNA nanopores exhibited cytotoxic effects [Fig. 7(c)],^145^ showcasing the potential of using DNA based channels as anticancer agents in biomedical applications.

To sum up, incorporation of hydrophobic groups onto surfaces of DNA nanopores is a key process to lower the energy barrier for the stable insertion of synthetic nanopores into lipid membranes. Virtually any modification can be spatially controlled and conjugated to DNA nanopores, bringing more promising capabilities in the near future. This is yet again a field that has significantly grown over the past years. There have been excellent reviews written on the topic.^203,204^

### F. Opportunities once the cellular membrane has been bypassed

We have shown the capability of DNA nanotechnology to interact with cellular membranes in biomimetic and highly controllable ways. One benefit of membrane-interfacing DNA nanostructures is their ability to deliver other DNA-based tools for sensing purposes to desired locations within a cell. In this section we briefly describe two such applications that allow quantitative biological imaging and biomolecular sensing.

**FIG. 8.**
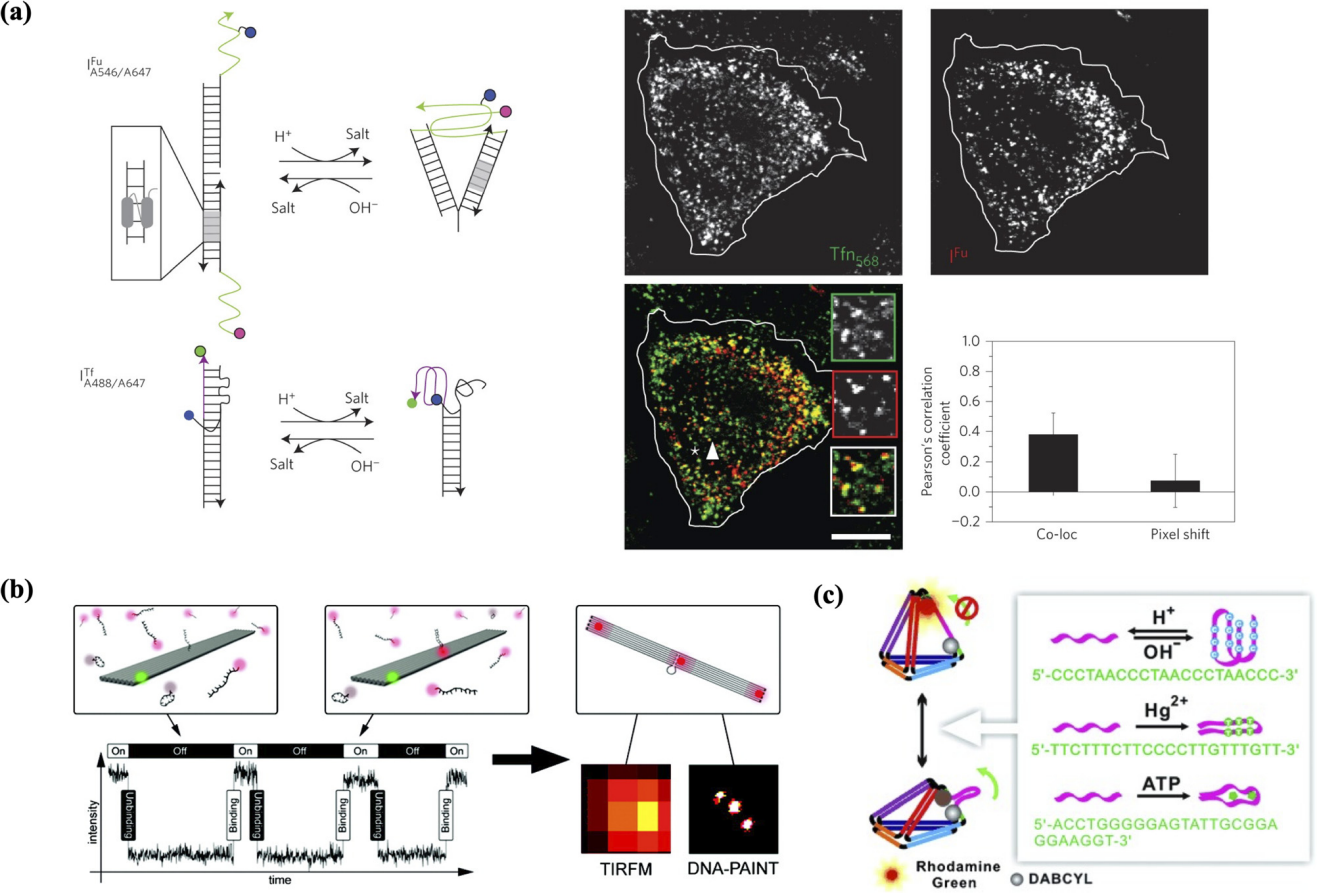
In-cell DNA nanostructures: (a) DNA nanomachines map pH changes in defined intracellular organelles inside the same cell along two different but intersecting cellular entry pathways, furin (Fu) and transferrin (Tf) pathways. pH-dependent conformational changes lead to fluorescence signal changes. Reprinted by permission from Modi *et al.*, Nat. Nanotechnol. **8**, 459 (2013).^205^ Copyright 2013 Springer Nature. (b) DNA point accumulation for imaging in nanoscale topography (DNA-PAINT) uses fluorescently labeled imager strands (red) and a DNA origami breadboard incorporating a fluorescent staple strand (green) and docking strands. Hybridization and dissociation of docking and imager strands result in ON and OFF of the fluorescence signal, which is monitored by total internal reflection fluorescence microscopy (TIRFM).^206^ Reprinted with permission from Jungmann *et al.*, Nano Lett. **10**, 4756 (2010). Copyright 2010 American Chemical Society. (c) Reconfigurable DNA tetrahedral nanosensors with a FRET-labeled probe sequence detect protons, ${\text{Hg}}^{2+}$, ATP, and complementary ssDNA through conformational changes.^207^ Reproduced with permission from Pei *et al.*, Angew. Chem., Int. Ed. **51**, 36 (2012). Copyright 2012 Authors, licensed under a Creative Commons Attribution (CCBY) License.

DNA-mediated quantitative imaging has seen profound advancement in recent years. As an example of the capabilities achieved, in a study two DNA nanomachines labeled with fluorophores were designed to map pH changes in defined intracellular organelles by taking different endocytic pathways with furin and transferrin as the mediating receptors respectively. Conformational change of nanomachines under different pH environments led to fluorescence signal changes [Fig. 8(a)].^205^ Regardless of whether they were delivered sequentially or simultaneously, both DNA nanomachines were able to capture the pH of early endosomes and trans-Golgi network. In another study by the Krishnan group, a DNA-based fluorescent reporter, CalipHluor, was developed to simultaneously record both pH and ${\text{Ca}}^{2+}$ in targeted acidic organelle through the scavenger receptor-mediated endocytic pathway.^208^ CalipHluor overcame the long-standing difficulty caused by the pH sensitivity of ${\text{Ca}}^{2+}$ reporters. The pH corrected ${\text{Ca}}^{2+}$ map was then constructed with the affinity map, which was computed from reporter affinity in the pH map, and the object-relational (O/R) map. With this technique, lumenal ${\text{Ca}}^{2+}$ as a function of endosomal maturation was mapped and used to measure how *catp-6*, a *Caenorhabditis elegans* homolog of ATP13A2, facilitated lysosomal ${\text{Ca}}^{2+}$ accumulation. In addition, the DNA-PAINT technique [Fig. 8(b)],^206,209^ along with other DNA-based nanodevices that utilize Förster resonance energy transfer (FRET) or fluorophore-quencher imaging system have been shown to have great potential as tools to image biological systems. For more discussions on DNA nanodevices in biological imaging, we refer the reader to a detailed review by Chakraborty *et al.*^28^

In biomolecular sensing, multiple reconfigurable DNA tetrahedral nanosensors were constructed to be responsive to protons, ${\text{Hg}}^{2+}$, ATP, complementary ssDNA [Fig. 8(c)],^207^ mRNA,^210,211^ pH and superoxide anion concentration^212^ *in vitro* and *in vivo*. However, for these in-cell biomolecular nanosensors, their cellular uptake pathways were not exactly clear. We posit that DNA nanosystems are uniquely suited to investigate the “rules” for triggering cellular uptake. With an improved understanding of uptake pathways, the rationale design and therefore the internalization efficiency and detection sensitivity of these systems can be further enhanced.

## VI. CONCLUSIONS

The high level control of size and shape, the programmable conformational change and corresponding external stimulus-responsive structure dynamics, and spatially well-defined decoration with a plethora of functional moieties have given nucleic acid-based nanostructures the potential to build nanomaterials and nanomachines with desired biological, chemical and biomechanical functionalities. These properties have the potential to enable powerful new applications in the study and manipulation of cell membranes.

We have aimed to introduce major opportunities and challenges for structural DNA nanotechnology applications in this interdisciplinary endeavor. By providing a detailed introduction to the mechanics of the membrane, we hope to equip readers with the knowledge needed to develop new applications of DNA nanostructures interfacing with the lipid membranes *in vitro* and *in vivo*. First of all, it is necessary for functional DNA nanostructures to maintain stability and integrity over an extended period of time, before and after they are delivered to target positions, under physiological conditions. Second, understanding how DNA nanostructures talk to lipid membranes, especially cellular membranes, and the governing mechanisms of the interactions between DNA nanostructures and lipid membranes, are still not clear. More fundamental and quantitative research is needed to improve the rational design and manufacturing of DNA nanostructures, promoting robust attachment to the membranes or efficient delivery. For instance, researchers must explore methods to minimize, or even stop if possible, cellular uptake of DNA nanostructures when aiming to place them on the membrane's exterior surface. Furthermore, although the technical barrier and cost of designing and constructing these structures have been continuously lowered by recent rapid advancements in DNA synthesis, functionalization of DNA oligos remains expensive.

The field has been attracting substantial research recently with efforts being dedicated to the emerging need at the interface of DNA nanostructures and cellular membranes. These investigations require interdisciplinary teams and highly collaborative works. Encouragingly, we are seeing the formation of a unique community where researchers from different backgrounds including biology, chemistry, physics and engineering, contribute and set foundations for utilizing nucleic acid-based nanostructures to probe, manipulate and explore membrane systems.

## AUTHORS' CONTRIBUTIONS

W.W. and D.S.A contributed equally to this work.

## Data Availability

Data sharing is not applicable to this article as no new data were created or analyzed in this study.
